# Phylogeny and Biogeography of the Carnivorous Plant Family Sarraceniaceae

**DOI:** 10.1371/journal.pone.0039291

**Published:** 2012-06-13

**Authors:** Aaron M. Ellison, Elena D. Butler, Emily Jean Hicks, Robert F. C. Naczi, Patrick J. Calie, Charles D. Bell, Charles C. Davis

**Affiliations:** 1 Harvard Forest, Harvard University, Petersham, Massachusetts, United States of America; 2 Organismic and Evolutionary Biology, Harvard University, Massachusetts, United States of America; 3 Biological Sciences, Eastern Kentucky University, Richmond, Kentucky, United States of America; 4 Regulatory Services, University of Kentucky, Lexington, Kentucky, United States of America; 5 The New York Botanical Garden, Bronx, New York, United States of America; 6 University of New Orleans, New Orleans, Louisiana, United States of America; CNRS/Université Joseph-Fourier, France

## Abstract

The carnivorous plant family Sarraceniaceae comprises three genera of wetland-inhabiting pitcher plants: Darlingtonia in the northwestern United States, Sarracenia in eastern North America, and Heliamphora in northern South America. Hypotheses concerning the biogeographic history leading to this unusual disjunct distribution are controversial, in part because genus- and species-level phylogenies have not been clearly resolved. Here, we present a robust, species-rich phylogeny of Sarraceniaceae based on seven mitochondrial, nuclear, and plastid loci, which we use to illuminate this family's phylogenetic and biogeographic history. The family and genera are monophyletic: *Darlingtonia* is sister to a clade consisting of *Heliamphora+Sarracenia*. Within *Sarracenia*, two clades were strongly supported: one consisting of *S. purpurea*, its subspecies, and *S. rosea*; the other consisting of nine species endemic to the southeastern United States. Divergence time estimates revealed that stem group Sarraceniaceae likely originated in South America 44–53 million years ago (Mya) (highest posterior density [HPD] estimate = 47 Mya). By 25–44 (HPD = 35) Mya, crown-group Sarraceniaceae appears to have been widespread across North and South America, and *Darlingtonia* (western North America) had diverged from *Heliamphora*+*Sarracenia* (eastern North America+South America). This disjunction and apparent range contraction is consistent with late Eocene cooling and aridification, which may have severed the continuity of Sarraceniaceae across much of North America. *Sarracenia* and *Heliamphora* subsequently diverged in the late Oligocene, 14–32 (HPD = 23) Mya, perhaps when direct overland continuity between North and South America became reduced. Initial diversification of South American *Heliamphora* began at least 8 Mya, but diversification of *Sarracenia* was more recent (2–7, HPD = 4 Mya); the bulk of southeastern United States *Sarracenia* originated co-incident with Pleistocene glaciation, <3 Mya. Overall, these results suggest climatic change at different temporal and spatial scales in part shaped the distribution and diversity of this carnivorous plant clade.

## Introduction

Carnivory has evolved at least six times within the flowering plants [1], [2] and is thought to be an adaption to increase the uptake of nitrogen and phosphorous in the nutrient-poor, aquatic and wetland environments where these plants grow [3], [4]. The biogeographic distribution of carnivorous plants presents as intriguing a puzzle as the evolution of carnivory itself, but far more attention has been directed at understanding the evolution of carnivorous plants [2], [3], [5] than has been directed at understanding their biogeography. Here, we present the most fully-resolved phylogeny of the American pitcher-plant family Sarraceniaceae to date. We use these data to estimate molecular divergence times of the group and to address a long-standing debate on the biogeographic origin and the disjunct distribution of these three genera.

**Figure 1:**
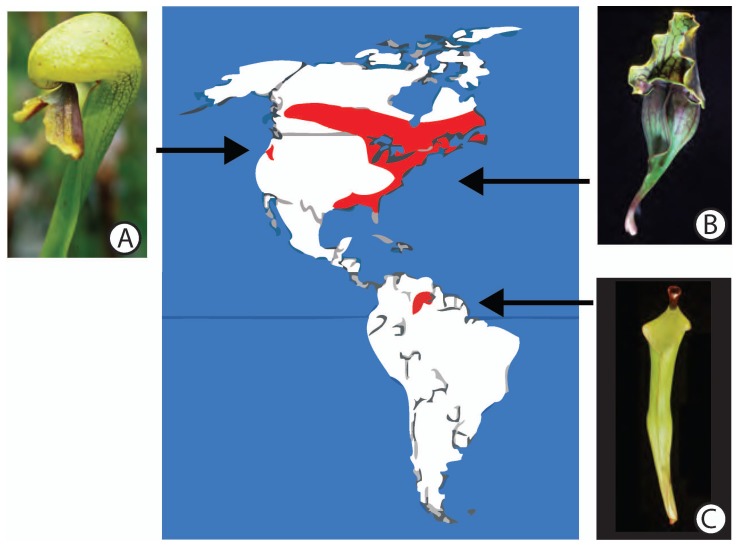
Geographic distribution of Sarraceniaceae. *Darlingtonia* (A) is restricted to western North America, *Sarracenia* (B) is widespread in Eastern North America, and *Heliamphora* (C) occurs in northern South America [17], [27]. Photographs by the authors.

Carnivorous plants grow on every continent except Antarctica. Some carnivorous plant families, such as the Cephalotaceae, Roridulaceae, and Byblidaceae, are endemics occurring on single (sub)continents, whereas others, such as Droseraceae and Lentibulariaceae have cosmopolitan distributions [1], [2], [5]–[11]. The enigmatic, disjunct distribution of the three genera of the American pitcher plants, Sarraceniaceae (Fig. 1), presents an unresolved question for botanists, biogeographers, and evolutionary biologists. Sarraceniaceae includes at least 30 species in three genera: one species of *Darlingtonia* Torr., 11 species of *Sarracenia* L., and at least 18 species of *Heliamphora* Benth. Sarraceniaceae itself is a well-supported member of the Ericales [2], [12]–[15], and is distinguished from other close relatives by its modified pitcher-like leaves [16] that trap and digest arthropod prey [17], and nodding, bisexual flowers [14] that are pollinated by a variety of bees and flies [18]–[20].

The single species of *Darlingtonia*, *D. californica* Torr., is endemic to the serpentine seeps and interdunal wetlands of northern California and southwestern Oregon in western North America [14], [21]. All of the species in the tropical genus *Heliamphora* grow atop sandstone massifs (tepuis) and nearby savannas in the Guayana Highlands of Venezuela, Guyana, and Brazil [22]–[25], where the spatial separation of these tepuis is thought to have led to diversification through allopatric speciation [24], [25]. The genus *Sarracenia* ranges from the Gulf Coast of Texas, Louisiana, Mississippi, Alabama, and Florida, north along the Atlantic Coast to Newfoundland and Labrador, and west through the northern Midwestern United States and southern Canada to eastern British Columbia [14], [26], [27]. All eleven species of *Sarracenia* [14] can be found, often growing sympatrically and readily hybridizing, in the southeastern United States, but only one, *S. purpurea* L. ssp. *purpurea* (Raf.) Wherry, grows in the northern regions of North America that were glaciated during the Pleistocene [26], [27]. Presently, *Sarracenia purpurea* spp. *purpurea* has a nearly transcontinental range, but the remaining species have much smaller ranges. Three centuries of habitat fragmentation and outright destruction, along with extensive legal and illegal collecting of these plants, however, makes assessing their “contemporary” ranges difficult.

At least five hypotheses have been proposed to explain the disjunct distribution of Sarraceniaceae [28]. The first four hypotheses emphasize the role of dispersal and posit a single center of origin for the family, either in tropical South America [24], [29] or in southeastern North America [30]. Croizat [6] and McDaniel [31] proposed two of the dispersal hypotheses, and suggested that Sarraceniaceae is an ancient lineage; its present distribution in eastern and western North America arose from two independent, Cretaceous-era dispersal events from South American ancestors. Gleason presented an alternative hypothesis: dispersal to North America occurred very recently during the Pleistocene, first via the Antillean Arc to southeastern North America, and second from southeastern North America to the Pacific Northwest (H. A. Gleason *pers. comm*. 1969 to B. Maguire, *fide* [24]). The final dispersal hypothesis is that the family originated in what is now southeastern North America during the Eocene (∼40–60 Mya), and achieved its present distribution via two dispersal events: one into northwest North America and the other into northern South America [30].

The fifth hypothesis emphasizes vicariance associated with climatic change [18]. Renner hypothesized that species in this family were once widely distributed across present-day North and South America, but she did not specify the time or location for the origin of the family. She then concluded that the present disjunct distribution of Sarraceniaceae arose as a result of fragmentation of this once more widespread range due to climatic changes that sharply reduced the areal extent of their acidic, boggy habitats (although these habitats themselves were likely patchily distributed across the Americas [22]). Such climatic changes are thought to have occurred during end-Eocene/Oligocene cooling (∼35–50 Mya [32]) and again during the Pleistocene glaciation and interglacials (∼2.6 Mya – 11.5 kya; [32]–[34]).

A better understanding of the phylogenetic relationships within Sarraceniaceae can help distinguish among these competing biogeographic hypotheses. Previous studies using plastid (cp) *rbcL* [1], [22] and nuclear (nu) ribosomal ITS and 26S rRNA sequence data [22], [28] supported similar phylogenetic relationships for the clade. All three genera were resolved as monophyletic, and *Darlingtonia* is placed as sister to the *Sarracenia*+*Heliamphora* clade. Not all of these studies, however, sampled broadly within the species-rich genera *Sarracenia* and *Heliamphora*. Furthermore, those that sampled multiple species achieved relatively little phylogenetic resolution within these genera [22], [28].

Here, we used cp, nu, and mitochondrial (mt) sequence data to resolve the phylogeny of Sarraceniaceae. Ours is the first study to include not only representatives from all three genera of Sarraceniaceae, but also complete species-level sampling for *Sarracenia*, including multiple accessions of the *S. purpurea* and *S. rubra* complexes, which have been described at different times as distinct species, subspecies, or varieties [14]. We then use these data to estimate molecular divergence times and ancestral ranges to infer the biogeographic history of this enigmatic plant clade. Results from our study also may help to explain the biogeography of other similarly distributed groups, such as *Clintonia* (Liliaceae), *Trillium* (Trilliaceae), and other forest herbs that exhibit high diversity in southeastern North America, low diversity in northeastern North America, and also occasional disjuncts in western North America [34], [35].

## Results

### Phylogenetic analyses

**Figure 2:**
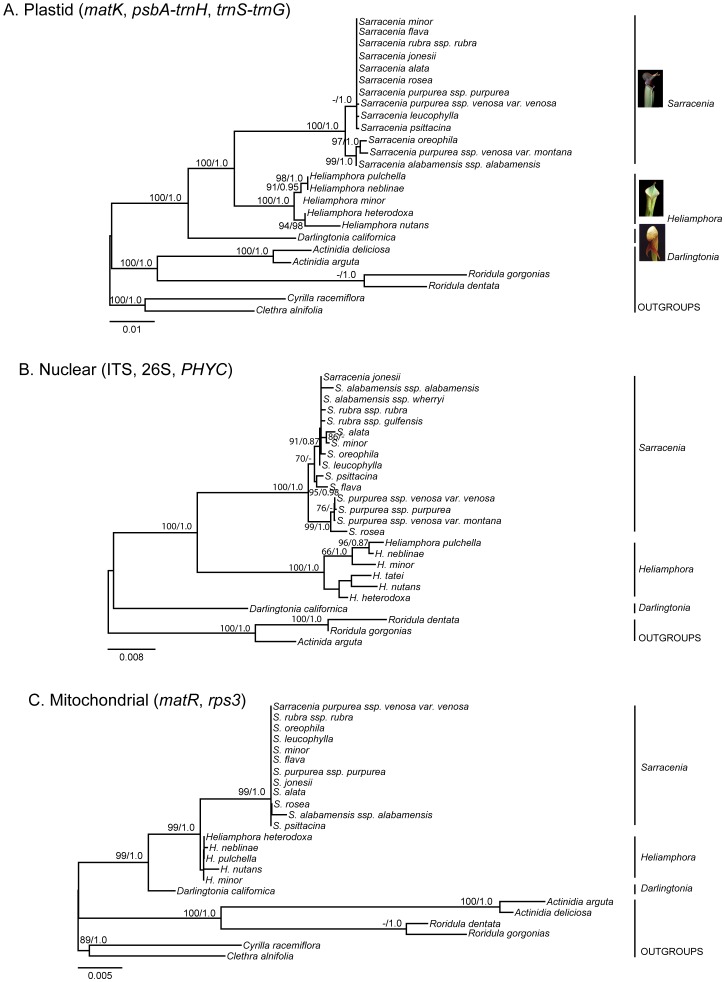
Maximum likelihood phylogenies of Sarraceniaceae. Phylogenies are based on (**A**) plastid (*matK*, *psbA-trnH*, *trnS-trnG*); (**B**) nuclear (ITS, 26S, *PHYC*); and (**C**) mitochondrial (C, *matR, rps3*) sequence data. ML bootstrap percentages >65 and Bayesian posterior probabilities >0.85 are indicated at the nodes, respectively. Scale bar shows nucleotide substitutions per site.

**Figure 3:**
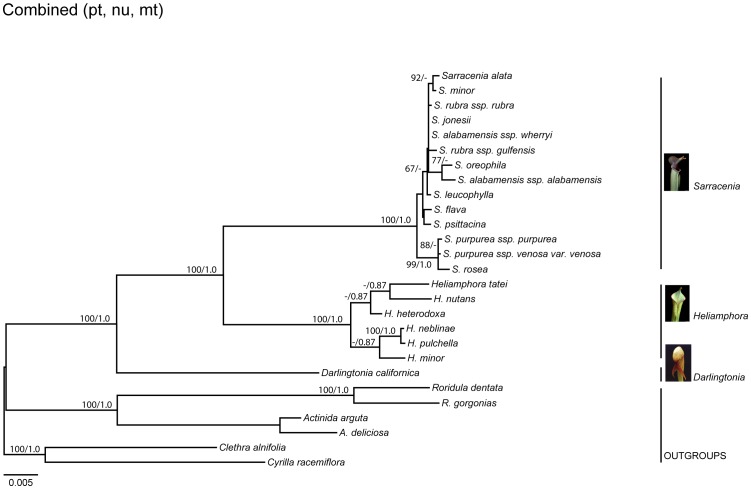
Maximum likelihood phylogeny of Sarraceniaceae based on plastid, nuclear, and mitochondrial data combined. *Sarracenia purpurea* var. *montana* was excluded from this analysis (see text). ML bootstrap percentages >65 and Bayesian posterior probabilities >0.85 are indicated at the nodes, respectively. Scale bar shows nucleotide substitutions per site.

Our aligned nu [ITS, 26S, *PHYC*], cp [*matK, psbA-trnH*, *trnS-trnG*], and mt [*matR, rps3*] datasets included 4463, 2317, and 2846 nucleotide base pairs, respectively. All analyses (Figs. 2, 3) supported the monophyly of Sarraceniaceae and each of the three genera in the family, *Darlingtonia*, *Sarracenia*, and *Heliamphora,* with very high support (100 percent bootstrap support [BS]; 1.0 Bayesian posterior probability [PP]). Within Sarraceniaceae, *Heliamphora* always emerged as sister to *Sarracenia* (Figs. 2, 3). Different samples identified as the same taxon (Table S1) based on morphology were consistently identified as the same taxon using sequence data.

The cp and nu phylogenies (Figs. 2A, B, respectively) were largely congruent with one conspicuous exception: the cp phylogeny did not place *S. purpurea* ssp. *venosa* var. *montana* D.E. Schnell & Determann with other members of the *S. purpurea* complex; instead, in the cp phylogeny this variety was well-supported (97 BS; 1.0 PP) as sister to *S. oreophila* Wherry. This possible instance of chloroplast capture involving *S. purpurea* ssp. *venosa* var. *montana* merits additional investigation. In the cp phylogeny, the subclade consisting of *S. purpurea* ssp. *venosa* var. *montana*+*S. oreophila* in turn was sister to *S. alabamensis* Case & R.B. Case ssp. *alabamensis* (99 BS; 1.0 PP).

In the nu phylogeny, the *S. purpurea* complex (the two subspecies of *S. purpurea*+*S. rosea*) was very well supported (99 BS; 1.0 PP; Fig. 2B) as a clade, which is consistent with morphological hypotheses of relationships [28], [36]. In the *S. purpurea* clade itself, the more southerly distributed *S. rosea* Naczi, Case & R.B. Case was sister to a moderately supported (76 BS; <0.85 PP), more northerly distributed, clade that included *S. purpurea* ssp. *venosa* (Raf.) Wherry, *S. purpurea* ssp. *venosa* var. *montana*, and *S. purpurea* ssp. *purpurea* (Fig. 2B). The *S. purpurea* complex in turn was sister to a moderately supported (70 BS; <0.85 PP) clade containing the remaining *Sarracenia* species (Figs. 2B). In the clade of the remaining *Sarracenia* species, *S. psittacina* Mich. and *S. flava* L. formed a well-supported (95 BS; 0.98 PP) clade that was sister to a well-supported (91 BS; 0.87 PP) clade containing the remaining *Sarracenia* species: *S. alata* (Wood) Wood, *S. alabamensis* ssp. *alabamensis*, *S. jonesii* Wherry, *S. leucophylla* Raf., *S. minor* Walter, *S. oreophila*, and *S. rubra* Walt. (*sensu stricto*). Relationships of the latter species were largely unresolved, but a clade containing *S. alata* and *S. minor* was moderately supported (86 BS; <0.85 PP).

In *Heliamphora*, relationships were generally well-supported and identical between the cp and nu phylogenies (Figs. 2A, B). *Heliamphora pulchella* Wistuba, Carow, Harbarth & Nerz and *H. neblinae* Maguire formed a well-supported clade (>95 BS; 1.0 PP) that was sister to *H. minor* Gleason (91 BS, 1.0 PP in the cp phylogeny [Fig. 2A]; 66 BS, 1.0 PP in the nu phylogeny [Fig. 2B]). This clade was, in turn, sister to a sub-clade including *H. heterodoxa* Steyerm. and *H. nutans* Benth (94 BS; 0.98 PP in the cp phylogeny [Fig. 2A]; <60 BS, <0.60 PP in the nu phylogeny [Fig. 2B]). In the nu phylogeny, we also included *H. tatei* Gleason, which grouped as sister to *H. nutans* but without strong statistical support (<50 BS, <0.5 PP). When this taxon was removed, support values in the nu phylogeny all increased to >90 BS, >0.95 PP (results not shown). This suggests that although there was a very high degree of congruence between the two topologies, this taxon may be the cause of the overall drop in support values observed between the cp and nu phylogenies.

The mt phylogeny (Fig. 2C) produced no additional resolution within either *Sarracenia* or *Heliamphora*.

Based on this apparently strong topological conflict between the nu and cp phylogenies (Fig. 2A–B), we removed *S. purpurea* ssp. *venosa* var. *montana* from the combined analysis. Our combined phylogeny of the remaining taxa based on the cp, nu, and mt data was well-supported (>85 BS, >0.85 PP, except for the southeastern U.S. *Sarracenia* subclade; Fig. 3) and consistent with relationships inferred from our individual gene trees (Fig. 2). Well-supported (>85 BS; >0.95 PP) relationships were largely consistent with the nu phylogeny, but the overall support was less in the combined tree than in the nu tree alone. The one exception was within *Sarracenia*: *S. alata*+*S. minor*, which were weakly supported as a clade in the nu tree, received high BS support (92 BS, but <0.85 PP) in the combined analysis. Additionally, *S. oreophila* was identified as a moderately supported (77 BS; <0.85 PP) sister to *S. alabamensis* ssp. *alabamensis*, mirroring the cp analysis.

### Topological tests

All alternative tree constrained topologies reflecting rival biogeographic explanations of Sarraceniaceae were determined to be significantly worse (*P*<0.005) explanations of the data than the unconstrained ML tree (Fig. 3) based on the approximately unbiased (AU) test.

### Molecular divergence time estimates

**Figure 4:**
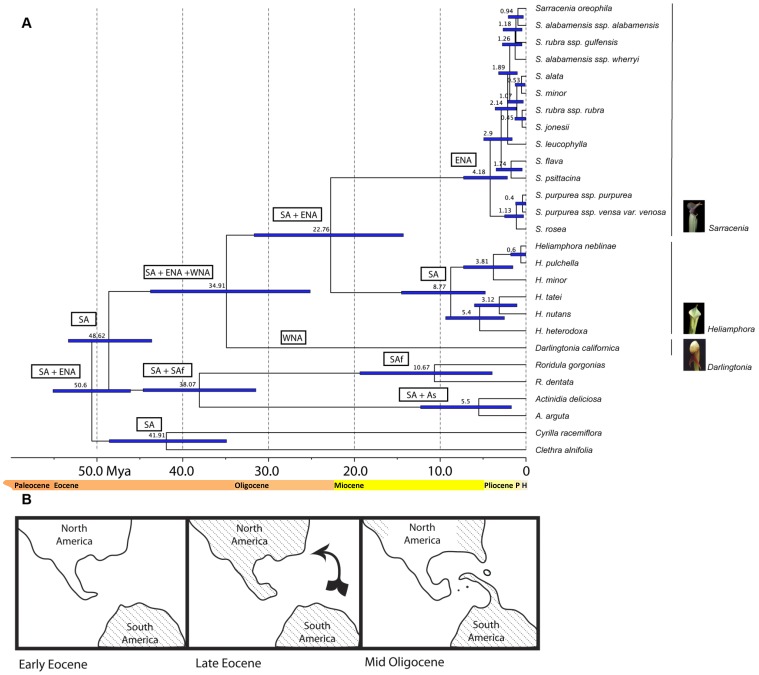
BEAST chronogram for the combined data and hypothesized biogeographic history of Sarraceniaceae. (A) Mean divergence times estimates are shown at the nodes of the cladogram. 95% posterior probability distribution shown with thick blue lines. Ancestral areas reconstructions from LAGRANGE [70], [71] shown in boxes near nodes. SA = South America; ENA = Eastern North America; WNA = Western North America; SAf = South Africa; and As = Asia. (B) We hypothesize that Sarraceniaceae originated in the Middle Eocene, perhaps in South America, and achieved its widespread distribution in North and South America by the Late Eocene. An early migration of Sarraceniaceae out of South America during the Eocene may have been facilitated via land connections in the proto-Caribbean. This connection would likely have been unavailable for direct overland migration by the mid-Oligocene, which is consistent with the early Oligocene disjunction of northern (*Sarracenia*, *Darlingtonia*) and southern (*Heliamphora*) members of Sarraceniacace. An East (*Sarracenia*+*Heliamphora*)/West (*Darlingtonia*) disjunction occurred in the very latest Oligocene, and may have been attributable to broad scale cooling and aridification during the late Oligocene.

Our mean nodal Bayesian divergence time estimates (Fig. 4A) indicate that stem-group Sarraceniaceae originated by the Middle Eocene, ∼47 Mya (95% highest posterior density [HPD]: 44–53 Mya). Within crown-group Sarraceniaceae, *Darlingtonia* diverged from *Heliamphora*+*Sarracenia* in the Late Eocene, ∼35 Mya (HPD: 25–44 Mya); and *Heliamphora* and *Sarracenia* diverged from one another in the Late Oligocene, 23 Mya (HPD: 14–32 Mya). *Heliamphora* began to diversify during the Late Miocene, 9 Mya (HPD: 5–14 Mya). *Sarracenia* was the most recent clade to diversify during the Pliocene, 4 Mya (HPD: 2–7 Mya). The remaining two major subclades in *Sarracenia* (*S. purpurea*+*S. rosea*; the remaining species) diversified 1 (HPD: 0.5–2) and 3 (HPD: 2–5) Mya, respectively.

### Ancestral areas reconstructions

Our ancestral area reconstructions (Fig. 4) indicated that stem-group Sarraceniaceae most probably originated in South America and that species in crown-group Sarraceniaceae were widespread in South America, western North America, and eastern North America. The most recent common ancestor of *Heliamphora* and *Sarracenia* was likely present in South America and eastern North America, whereas *Darlingtonia* was restricted to western North America. Subsequently, the ancestor of *Heliamphora* and *Sarracenia* occurred in South America and Eastern North America and diverged into South American and Eastern North American subclades, respectively.

## Discussion

The phylogeny inferred from our analysis of cp, nu, and mt genes (Figs. 2, 3) provides the most fully resolved phylogeny of Sarraceniaceae to date. Our results support the consensus that all three genera are monophyletic and that *Darlingtonia* is sister to *Heliamphora*+*Sarracenia* [22], [28]. Our biogeographic analyses reveal that stem-group Sarraceniaceae originated in South America 44–53 Mya, and that by 25–44 Mya, crown-group Sarraceniaceae had achieved a widespread distribution across South and North America (Fig. 4A). Our new estimates of divergence times within and among clades (Fig. 4A) also provide support for the vicariance hypothesis proposed by Renner [18] to explain the biogeographic history of the family. Furthermore, our analyses are consistent with the hypothesis that multiple global climactic events, from more ancient cooling during the end of the Eocene [32], [34] to more recent Pleistocene glaciation [33], [34], may have shaped the biogeography and diversification of Sarraceniaceae. We first discuss the novel phylogenetic insights revealed by our analyses and then elaborate on our hypothesis regarding the biogeography and present-day distribution of the family.

### Novel relationships within *Sarracenia*

Our results provide clearer species-level resolution within *Sarracenia* than previous studies [22], [28]. In agreement with an earlier nu phylogeny [28], both our nu (Fig. 2A) and combined phylogeny (Fig. 3) support the placement of the *S. purpurea* complex as sister to the remaining species of *Sarracenia*, and also suggest that *S. rosea* is sister to the rest of the *S. purpurea* complex [28]. Within the remaining *Sarracenia* clade results are generally consistent with previous findings [22], [28]. The one exception is the placement of *S. minor*. In a previous study [28] this species was moderately placed with *S. psittacina* and *S. flava*. In contrast, we place it strongly in a subclade with *S. alata* (Fig. 3). Our finding that *S. psittacina* and *S. flava* are sister species does not support the separation of *Sarracenia* into species with prostrate pitchers (*S. psittacina* and the *S. purpurea* clade) versus those with upright pitchers (all remaining *Sarracenia* species) [37].

Relationships among the members of the *S. rubra* complex (including *S. jonesii*) remain incompletely understood from both a morphological and molecular standpoint [14], [28], and require further investigation. *Sarracenia rubra* ssp. *rubra* and *S. jonesii* are sister taxa in the cp phylogeny (Fig. 2A) and consistently group together in the BEAST analysis (Fig. 4), but support for this relationship is not strong in any of our analyses (Figs. 2, 3, 4). The lack of resolution within the *S. rubra* complex and other southeastern *Sarracenia* may be explained in part by the rapid diversification of the genus, and in part by the fact that *Sarracenia* species hybridize readily in the wild [28], [37], [38]. Indeed, Mellichamp [14] reports 19 known hybrids of wild origin. For example, it is possible that *S. alabamensis* ssp. *alabamensis*, *S. oreophila*, and *S. purpurea* ssp. *venosa* var. *montana*, which grow in near sympatry, arose through hybridization and introgression, and that this history of hybridization is still visible in the maternally-inherited genomes (Fig. 2A). Interestingly, our cp phylogeny (Fig. 2A) suggests that *S. purpurea* ssp. *venosa* var. *montana* may have inherited its plastid genome via chloroplast capture from these species, but shares its true species affinity with other members of the *purpurea* complex, which is supported by its placement in the nu phylogeny (Fig. 2B). Such a history of reticulation could explain the conflicting topologies of these taxa in the plastid and nuclear phylogenies.

### Relationships within *Heliamphora*

Our sampling of *Heliamphora* was limited – we sequenced only 6 of the 18 recognized taxa – but the relationships among the taxa we sampled were well-supported by both nu and cp data. The consensus tree (Fig. 3) supports the division of our taxa into two clades, one comprised of *H. neblinae*, *H. pulchella*, and *H. minor*, and one comprised of *H. tatei*, *H. nutans*, and *H. heterodoxa*. All six of these species grow on different tepuis separated by many kilometers of unfavorable intervening habitat. Given the much older age of the tepuis (Mesozoic Era erosion of the 1.6 Ga Roraima Supergroup craton [34], [39]), it is likely that alloptatric speciation occurred on these tepui “islands” [25]. The clades we found in our analyses (Figs. 2, 3, 4) differ somewhat from those found by Bayer et al. [22], in which *H. tatei* and *H. minor* formed a clade sister to *H. nutans*, but in all phylogenetic studies of this genus to date, there has not been sampling of all species in the genus. Ongoing systematic and phylogenetic work [40] should help resolve relationships within *Heliamphora*.

### Biogeography of Sarraceniaceae

We hypothesize that during the Eocene (∼34–56 Mya), Sarraceniaceae became widespread in the Americas perhaps by migrating from South to North America via a discontinuous landmass in the Antilles region that appears to have begun in the middle Eocene, ∼50 Mya [41] (Fig. 4B). Toward the end of the Eocene, land connections between South and North America are thought to have been fairly direct and appear to have facilitated the movement of several mammalian clades into the Antilles from South America [42], [43]. We note here that although seeds of modern-day *Sarracenia* disperse on average <10 cm [44], they (along with seeds of *Heliamphora* and *Darlingtonia*) are hydrophobic, and can disperse longer distances by skimming across water surfaces [22], [44]. Rare long-distance dispersal events of 1–10 m, combined with the rapid population growth rate of *Sarracenia* [45] could have led to its spread beyond 10,000 km within 15 million years.

By the end of the Eocene, Sarraceniaceae appears to have been widespread across North and South America. Once Sarraceniaceae became established in North America it appears to have spread across the continent, setting the stage for range fragmentation as the climate changed beginning in the Eocene. Indeed, during this time, ancestral populations in Western North America appear to have become severed from those in Eastern North America plus South America. The timing of this major disjunction corresponds roughly with the increasing cooling and drying of mid-continental North America that began in the Eocene (∼50 Mya) and ended in the early Oligocene (∼34 Mya [32], [34]). This sort of climactic shift would have been likely to dramatically affect Sarraceniaceae and other plants with similar distributions [27], [34].

The second hypothesized disjunction within Sarraceniaceae occurred in the Late Oligocene (∼23 Mya), and involved populations spanning South America and Eastern North America. Although some north-to-south connections were likely available between these regions during the late Eocene and into the Oligocene, it appears that nearly direct overland connections may have been broken by the time of this disjunction during the mid-Oligocene [46]. Thus, the subdivision of these land connections may have precipitated the disjunction between Sarraceniaceae of South America and Eastern North America (Fig. 4B).

It appears that the crown-group diversification of Eastern North American *Sarracenia* took place 2–7 Mya, with much of the diversification in the group taking place within the last 0.5–5 Mya. Under these circumstances it seems plausible that drying events driven by Pleistocene glaciation [33] may have spurred diversification and range expansion in this clade. The northward expansion of the *Sarracenia purpurea* complex from a more southern ancestor, as suggested by our phylogeny (Fig. 3), is compatible with the hypothesis that glaciation may have played an important role for the tempo and mode of diversification, range expansion and/or extinction in *Sarracenia*.

Finally, it is worth noting the contrasting pattern in the timing of diversification of North American *Sarracenia* versus South American *Heliamphora*. Our estimates for *Heliamphora* suggest that its crown group diversification of 5–14 Mya is nearly twice as old as the crown group diversification of *Sarracenia*. Our sampling for *Heliamphora* is, however, incomplete, and the actual time of its crown group diversification may be even older. Nevertheless, the observed differences imply different triggers in the diversification of *Heliamphora* and *Sarracenia*, respectively. Alternatively, this trend may represent more widespread extinction of Sarraceniaceae during the Pliocene. In the long term, linking paleocolimatic reconstructions [34], [47] with a better sampled phylogeny of the entire group that combines morphological and molecular data could help to resolve relationships within *Sarracenia* [48] and provide further insights into the biogeography of this unusual plant family.

## Materials and Methods

### Taxon sampling

We sampled 22 accessions of Sarraceniaceae (Table S1). These included the monotypic *Darlingtonia californica*, six of the 18 species of *Heliamphora*, and all 11 recognized species of *Sarracenia* [14]. In *Sarracenia* we included three accessions from the *purpurea* complex (ssp. *purpurea*, ssp. *venosa* var. *venosa*, and ssp. *venosa* var. *montana*), two accessions from the *S. rubra* complex (ssp. *gulfensis*, and ssp. *rubra*), and two accessions from *S. alabamensis* (ssp. *alabamensis*, and ssp. *wherryi*). *Roridula* (Roridulaceae), *Actinidia* (Actinidiaceae), and *Clethra* (Clethraceae) were included as outgroups [15]. Plants were obtained from the seed-grown research collection of *Sarracenia* at Harvard Forest, Petersham, Massachusetts, USA [49]; from the research collection of living *Sarracenia* species of Frederick W. Case, Jr. in Saginaw, Michigan, USA; from the private *Heliamphora* collections of Steve Boddy, Cliff Dodd, and Charles Powell; or from commercial growers (California Carnivores, Sebastopol, California, USA, and Meadowview Biological Research Station, Woodford, Virginia, USA). Roridulaceae tissues were obtained from the collections of the Ecology & Evolutionary Biology Plant Growth Facilities at the University of Connecticut, Storrs, Connecticut, USA. *Actinidia deliciosa* tissue was obtained from a store-bought kiwifruit and is unvouchered. Additional sequences of Sarraceniaceae [28] were obtained from GenBank (Table S1). No specific permits were required for the described field studies. Specifically, no permits were required for collecting seeds of *Sarracenia alata*, *S. flava*, *S. leucophylla*, *S. minor* plant no. 1 in Table S1, or *S. rubra* ssp. *rubra*, as these species were neither protected nor endangered, and permits for collecting seeds from these pitcher plants were not required by any state or the US Federal Government in 2001 when seeds were gathered. No permits were required for collecting leaf tissue of the common *Sarracenia purpurea* ssp. *purpurea* (plant no. 1 in Table S1) from land owned by Harvard Forest or in the state of Michigan (*S. purpurea* ssp. *purpurea* plant no. 3 in Table S1), as the plant is not regulated or listed as Threatened, Endangered, or of Special Concern in the states of Massachusetts or Michigan (USA). No permits were required for using leaf tissue obtained from plants grown in cultivation by commercial growers or by individual collectors (all other taxa).

### DNA amplification and sequencing

We sequenced three cp (*matK, psbA-trnH* and *trnS-trnG*), two mt (*matR, rps3*), and three nu (ITS, 26S, *PHYC*) DNA regions. DNA was extracted either from 0.5–1.0 grams of silica-dried leaf/floral tissue using the DNeasy Plant Mini Kit protocol (QIAGEN, Valencia, California, USA) or from 0.5–1.0 gram of fresh leaf material using the CTAB protocol [50].

Polymerase chain reaction (PCR) amplification and sequencing of *matK* used primers 400F and trnK2r [51]; matK1, matK6 and matK1506 [52]; 870F and 1750F (J. Panero, *pers. comm.*]; matK5 [53]; and SmatK3 [54]. The cp spacer regions *trnH-psbA* and *trnS-trnG* were amplified using published primers and protocols [53]. Amplification and sequencing of *matR* used primers 26F and 1858R [55] or primers matR3′R and matR5′F [56] and a touchdown PCR protocol [57]. Amplification and sequencing of *rps3* followed reference [58]. The 26S locus was amplified using the overlapping primer sets S1/2134rev and S8/3058rev [59]. Nuclear ITS was amplified using the primers ITS4 [60] and ITS-LEU [61]. We cloned ITS to assess sequence heterogeneity [62]. We screened up to eight clones for each accession to check for multiple copies. In the cases where we directly sequenced ITS amplicons, the chromatograms yielded non-overlapping peaks, suggesting that ITS was single copy. *PHYC* was amplified using the cdo and int1F primer pair [63] and a touchdown PCR protocol [57]. PCR amplicons were gel-extracted as above and fragments were purified using the Millipore Ultrafree-DA columns (Millipore Corporation, Bedford, Massachusetts, USA). Up to five *PHYC* clones were sequenced for each accession to test for multiple copies. Directly sequenced amplicons yielded non-overlapping eletropherograms, suggesting the *PHYC* was a single copy. This is consistent with previous studies of other plant lineages showing that PHYC is single-copy [63]–[65].

### Phylogenetic analyses

Nucleotide sequences were first aligned automatically using MAFFT [66] and then manually refined by eye using Se-Al v2.0a11 Carbon [67]. Maximum likelihood (ML) was implemented in RAxML 7.0.4 [68] using CIPRES [69]. ML bootstrap percentages (BS) were estimated from 1000 rapid bootstrapping replicates [67] and Bayesian posterior probabilities were obtained from BEAST [70]. The combined dataset was partitioned by locus and analyzed using the General Time Reversible model, with rate heterogeneity modelled by assuming that some sites are invariable and that the rate of evolution at other sites approximates a discrete gamma distribution [GTR+I+Γ]). This model was determined to be the best fitting based on a likelihood ratio test for the concatenated data, as well as for each of the individual partitions. ML trees were inferred by genome (mt, cp, nu) and for the combined dataset. Clethraceae and Cyrillaceae were included as additional outgroups for *matK* and *matR*; for the remaining genes, only *Roridula* (Roridulaceae) and *Actinidia* (Actinidiaceae) were used as outgroups. For the combined dataset, *Roridula* (Roridulaceae) and *Actinidia* (Actinidiaceae) were used as outgroups.

### Topological tests

To evaluate the rival biogeographic hypotheses that have been proposed for Sarraceniaceae, we constructed several constraint topologies and searched for optimal trees under these constraints using maximum likelihood. To test **Hypothesis 1**, that the distribution of Sarraceniaceae in eastern and western North America arose from two independent dispersal events from South American ancestors [6], [31], we constrained the exclusively South American *Heliamphora* clade to be non-monophyletic. To test **Hypothesis 2**, that dispersal of Sarraceniaceae occurred first via the Antillean Arc to southeastern North America and second from southeastern North America to the Pacific Northwest (H. A. Gleason *pers. comm*. 1969 to B. Maguire, *fide* [24]), we constrained the eastern North American *Sarracenia* and the northwestern North American *Darlingtonia* to be monophyletic. To test **Hypothesis 3**, that Sarraceniaceae achieved its present distribution in northwestern North America and South America via two dispersal events: one to the northwest and the other to the southeast [30], we constrained the eastern North American *Sarracenia* to be non-monophyletic. The hypothesis by Renner [18] was consistent with our biogeographic results, and therefore was not tested here.

All constrained searches were performed with PAUP* [71] with 100 replicates of random stepwise addition using TBR branch swapping. In the cases of **Hypotheses 1** and **3** the “converse” option was selected in PAUP* so that trees that did not meet the constraint were evaluated and retained. For example, for **Hypothesis 1** only trees in which *Heliamphora* was not monophyletic were evaluated. Optimal trees from each constraint search were then evaluated using the approximately unbiased test (AU) as implemented in CONSEL version 0.20 [72], [73].

### Divergence time estimation

A Bayesian Markov chain Monte Carlo (MCMC) approach to simultaneously estimate the phylogenetic history and divergence times of Sarraceniaceae was conducted using BEAST v.1.6.2 [70]. We combined the nu (16 taxa; 4468 aligned bp), cp (25 taxa; 2319 aligned bp), and mt (24 taxa; 2847 aligned bp) datasets. *Sarracenia purpurea* ssp. *venosa* var. *montana* was excluded from this combined analysis due to its strongly conflicting phylogenetic placement in the cp and nu phylogenies (see Results, above). We implemented a relaxed molecular clock (uncorrelated lognormal [74]) and a Yule tree prior. Since we had no complete set of sequences for any single accession, we merged sequences from different accessions of the same taxon to reduce the effects of missing data (Table S1).

Data were partitioned by genome and a GTR+I+Γ model with six rate categories was applied to each partition with base frequencies estimated from the data. Because several accessions were missing sequence data for some of the regions, clock models were linked across the partition in order to anchor these taxa. A *Sarracenia* fossil has been reported [75] but its ancient Cretaceous age (ca. 110 Mya) is much older than any previous estimates for Sarraceniaceae, or for most other Ericales, which includes this family [76]. Moreover, its origin in China is far outside of the present range of Sarraceniaceae. Due to the exceptionally ancient age of this fossil, and its geographic location relative to present-day distribution of this clade, we instead used a series of secondary age constraints from an angiosperm-wide analysis that relied on 21 fossil constraints [76]. The following constraints were applied with a normal prior distribution that spanned the full range of nodal age estimates: the most recent common ancestor (MRCA) of Actinidiaceae, Clethraceae, Cyrillaceae, Roridulaceae, Sarraceniaceae was set to 50 Mya (sd = 3 Mya); the MRCA of Clethraceae and Cyrillaceae was set to 42 Mya (4 Mya); the MRCA of Actinidiaceae and Roridulaceae was set to 44 Mya (5 Mya); and stem group Sarraceniaceae was set to 48 Ma (4 Mya) [76]. MCMC chains were run for 50 million generations, sampling every 1000 generations. Of the 50001 posterior trees, we excluded the first 1000 as burn-in. Mixing of the MCMC chain was checked using Tracer v.1.5 [70].

### Ancestral area reconstructions

Ancestral area reconstructions were conducted in a likelihood framework using the dispersal-extinction-cladogenesis model as implemented in LAGRANGE_cpp ver. 0.1 BETA2, applying a uniform weighting of area connectivity [77], [78]. Our input topology was a 10 000-tree subsample taken from the output of the BEAST analysis described above. Five areas of endemism consistent with the present distribution of our outgroup and ingroup sampling were specified for this analysis (Table S1): South Africa, East Asia, South America, Eastern North America, and Western North America. We did not restrict the maximum number of ancestral areas.

## Supporting Information

Table S1 **Taxa of Sarraceniaceae (** ***Darlingtonia, Heliamphora*** **, and** ***Sarracenia*** **species) and outgroups (** ***Actinidia*** **,** ***Clethra*** **,** ***Cyrilla*** **, and** ***Roridula*** **species) used in the phylogenetic analysis and ancestral area reconstruction of the family.** All sequences have been deposited in GenBank and vouchers are accessed as noted (CONN – University of Connecticut Herbarium; GH – Gray Herbarium, Harvard University). A sequence for which the voucher is a GenBank number is a previously published sequence that is also used in the analyses presented in this paper. Abbreviations for modern-day distributions are: EA – East Asia; ENA – Eastern North America; SAm – South America; SAf – South Africa; WNA – Western North America.
(DOC)
