# Supplementary material for: Phylogeny and Biogeography of the Carnivorous Plant Family Sarraceniaceae
Source: PLoS One. 2012 Jun 13;7(6):e39291. doi: 10.1371/journal.pone.0039291 (PMC3374786; doi:10.1371/journal.pone.0039291)
Supplement: Table S1 — Taxa of Sarraceniaceae ( Darlingtonia, Heliamphora , and Sarracenia species) and outgroups ( Actinidia , Clethra , Cyrilla , and Roridula species) used in the phylogenetic analysis and ancestral area reconstruction of the family. All sequences have been deposited in GenBank and vouchers are accessed as noted (CONN – University of Connecticut Herbarium; GH – Gray Herbarium, Harvard University). A sequence for which the voucher is a GenBank number is a previously published sequence that is also used in the analyses presented in this paper. Abbreviations for modern-day distributions are: EA – East Asia; ENA – Eastern North America; SAm – South America; SAf – South Africa; WNA – Western North America. (DOC) [file pone.0039291.s001.doc]

**Table S1**

| **Taxon** | **Distribution** | **Sequenced region** | **GenBank number** | **Voucher inforfmation** |
| --- | --- | --- | --- | --- |
| *Actinidia arguta* | EA | *matR* | GBANK-AF420991.1 | GBANK-AF420991.1 |
| *Actinidia arguta* | EA | *matK* | GBANK-AF322596.1 | GBANK-AF322596.1 |
| *Actinidia deliciosa* | EA | *matR* | GBANK-JQ218283 | no voucher; store-bought kiwifruit collected by E. Butler in Cambridge, MA |
| *Actinidia deliciosa* | EA | *PHYC* | GBANK-JQ218226 | no voucher; store-bought kiwifruit collected by E. Butler in Cambridge, MA |
| *Actinidia deliciosa* | EA | *PHYC* | GBANK-JQ260418 | no voucher; store-bought kiwifruit collected by E. Butler in Cambridge, MA |
| *Actinidia deliciosa* | EA | *psbA-trnH* | GBANK-JQ218228 | no voucher; store-bought kiwifruit collected by E. Butler in Cambridge, MA |
| *Actinidia deliciosa* | EA | *rps3* | GBANK-JQ218227 | no voucher; store-bought kiwifruit collected by E. Butler in Cambridge, MA |
| *Actinidia deliciosa* | EA | *trnS-trnG* | GBANK-JQ218229 | no voucher; store-bought kiwifruit collected by E. Butler in Cambridge, MA |
| *Actinidia deliciosa* | EA | *matK* | GBANK-JQ218264 | no voucher; store-bought kiwifruit collected by E. Butler in Cambridge, MA |
| *Clethra alnifolia* | SAm | *matK* | GBANK-AJ429281.1 | GBANK-AJ429281.1 |
| *Clethra alnifolia* | SAm | *matR* | GBANK-AF520204.1 | GBANK-AF520204.1 |
| *Cyrilla racemiflora* | SAm | *matK* | GBANK-AF380080.1 | GBANK-AF380080.1 |
| *Cyrilla racemiflora* | SAm | *matR* | GBANK-AY725892.1 | GBANK-AY725892.1 |
| *Darlingtonia californica 1* | WNA | *psbA-trnH* | GBANK-JQ218316 | Butler 20 (GH); cultiv. plant from California Carnivores, Sebastopol, CA |
| *Darlingtonia californica 1* | WNA | *rps3* | GBANK-JQ218297 | Butler 20 (GH); cultiv. plant from California Carnivores, Sebastopol, CA |
| *Darlingtonia californica 1* | WNA | *trnS-trnG* | GBANK-JQ218333 | Butler 20 (GH); cultiv. plant from California Carnivores, Sebastopol, CA |
| *Darlingtonia californica 1* | WNA | *matK* | GBANK-JQ218261 | Butler 20 (GH); cultiv. plant from California Carnivores, Sebastopol, CA |
| *Darlingtonia californica 2* | WNA | *26S* | GBANK-JQ519377 | Naczi 2659 (NY); U.S.A. California: Del Norte Co., cultiv. by F. Case |
| *Darlingtonia californica 2* | WNA | *matR* | GBANK-JQ619012 | Naczi 2659 (NY); U.S.A. California: Del Norte Co., cultiv. by F. Case |
| *Darlingtonia californica 3* | WNA | *matR* | GBANK-AY163751.1 | GBANK-AY163751.1 |
| *Heliamphora heterodoxa 1* | SAm | *matK* | GBANK-JQ218258 | Butler 17 (GH); cultiv. plant from California Carnivores, Sebastopol, CA |
| *Heliamphora heterodoxa 1* | SAm | *psbA-trnH* | GBANK-JQ218314 | Butler 17 (GH); cultiv. plant from California Carnivores, Sebastopol, CA |
| *Heliamphora heterodoxa 1* | SAm | *rps3* | GBANK-JQ218294 | Butler 17 (GH); cultiv. plant from California Carnivores, Sebastopol, CA |
| *Heliamphora heterodoxa 1* | SAm | *ITS* | GBANK-JQ218242 | Butler 17 (GH); cultiv. plant from California Carnivores, Sebastopol, CA |
| *Heliamphora heterodoxa 2* | SAm | *26S* | GBANK-JQ519386 | photograph of leaves from which DNA extr. (NY); cultiv. by S. Boddy |
| *Heliamphora heterodoxa 2* | SAm | *matR* | GBANK-JQ619021 | photograph of leaves from which DNA extr. (NY); cultiv. by S. Boddy |
| *Heliamphora minor* | SAm | *26S* | GBANK-JQ519383 | no voucher; cultiv. by C. Powell |
| *Heliamphora minor* | SAm | *matK* | GBANK-JQ619007 | no voucher; cultiv. by C. Powell |
| *Heliamphora minor* | SAm | *matR* | GBANK-JQ619018 | no voucher; cultiv. by C. Powell |
| *Heliamphora neblinae 1* | SAm | *matK* | GBANK-JQ218260 | Butler 19 (GH); cultiv. plant from California Carnivores, Sebastopol, CA |
| *Heliamphora neblinae 1* | SAm | *matR* | GBANK-JQ218280 | Butler 19 (GH); cultiv. plant from California Carnivores, Sebastopol, CA |
| *Heliamphora neblinae 1* | SAm | *PHYC* | GBANK-JQ260415 | Butler 19 (GH); cultiv. plant from California Carnivores, Sebastopol, CA |
| *Heliamphora neblinae 1* | SAm | *rps3* | GBANK-JQ218296 | Butler 19 (GH); cultiv. plant from California Carnivores, Sebastopol, CA |
| *Heliamphora neblinae 2* | SAm | *26S* | GBANK-JQ519388 | photograph of leaves from which DNA extr. (NY); cultiv. by C. Dodd |
| *Heliamphora neblinae 2* | SAm | *matR* | GBANK-JQ619023 | photograph of leaves from which DNA extr. (NY); cultiv. by C. Dodd |
| *Heliamphora nutans* | SAm | *26S* | GBANK-JQ519389 | no voucher; cultiv. by C. Powell |
| *Heliamphora nutans* | SAm | *matK* | GBANK-JQ619009 | no voucher; cultiv. by C. Powell |
| *Heliamphora nutans* | SAm | *matR* | GBANK-JQ619024 | no voucher; cultiv. by C. Powell |
| *Heliamphora pulchella* | SAm | *ITS* | GBANK-JQ218243 | Butler 18 (GH); cultiv. plant from California Carnivores, Sebastopol, CA |
| *Heliamphora pulchella* | SAm | *matK* | GBANK-JQ218259 | Butler 18 (GH); cultiv. plant from California Carnivores, Sebastopol, CA |
| *Heliamphora pulchella* | SAm | *matR* | GBANK-JQ218279 | Butler 18 (GH); cultiv. plant from California Carnivores, Sebastopol, CA |
| *Heliamphora pulchella* | SAm | *PHYC* | GBANK-JQ260414 | Butler 18 (GH); cultiv. plant from California Carnivores, Sebastopol, CA |
| *Heliamphora pulchella* | SAm | *psbA-trnH* | GBANK-JQ218315 | Butler 18 (GH); cultiv. plant from California Carnivores, Sebastopol, CA |
| *Heliamphora pulchella* | SAm | *rps3* | GBANK-JQ218295 | Butler 18 (GH); cultiv. plant from California Carnivores, Sebastopol, CA |
| *Roridula dentata* | SAf | *matK* | GBANK-JQ218262 | CONN 129998 (CONN); South Africa. Western Cape Prov., cultiv. Univ. Connecticut |
| *Roridula dentata* | SAf | *matR* | GBANK-JQ218282 | CONN 129998 (CONN); South Africa. Western Cape Prov., cultiv. Univ. Connecticut |
| *Roridula dentata* | SAf | *PHYC* | GBANK-JQ260417 | CONN 129998 (CONN); South Africa. Western Cape Prov., cultiv. Univ. Connecticut |
| *Roridula dentata* | SAf | *psbA-trnH* | GBANK-JQ218318 | CONN 129998 (CONN); South Africa. Western Cape Prov., cultiv. Univ. Connecticut |
| *Roridula dentata* | SAf | *rps3* | GBANK-JQ218299 | CONN 129998 (CONN); South Africa. Western Cape Prov., cultiv. Univ. Connecticut |
| *Roridula dentata* | SAf | *26S* | GBANK-JQ519390 | CONN 129998 (CONN); South Africa. Western Cape Prov., cultiv. Univ. Connecticut |
| *Roridula dentata* | SAf | *matR* | GBANK-JQ619025 | CONN 129998 (CONN); South Africa. Western Cape Prov., cultiv. Univ. Connecticut |
| *Roridula gorgonias* | SAf | *matK* | GBANK-JQ218263 | CONN 131684 (CONN); From seeds from the International Carnivorous Plant Society seedbank, cultiv. Univ. Connecticut |
| *Roridula gorgonias* | SAf | *matR* | GBANK-JQ218281 | CONN 131684 (CONN); From seeds from the International Carnivorous Plant Society seedbank, cultiv. Univ. Connecticut |
| *Roridula gorgonias* | SAf | *PHYC* | GBANK-JQ260416 | CONN 131684 (CONN); From seeds from the International Carnivorous Plant Society seedbank, cultiv. Univ. Connecticut |
| *Roridula gorgonias* | SAf | *psbA-trnH* | GBANK-JQ218317 | CONN 131684 (CONN); From seeds from the International Carnivorous Plant Society seedbank, cultiv. Univ. Connecticut |
| *Roridula gorgonias* | SAf | *rps3* | GBANK-JQ218298 | CONN 131684 (CONN); From seeds from the International Carnivorous Plant Society seedbank, cultiv. Univ. Connecticut |
| *Roridula gorgonias* | SAf | *trnS-trnG* | GBANK-JQ218334 | CONN 131684 (CONN); From seeds from the International Carnivorous Plant Society seedbank, cultiv. Univ. Connecticut |
| *Sarracenia alabamensis ssp. alabamensis 1* | ENA | *psbA-trnH* | GBANK-JQ218302 | Butler 9 (GH); cultiv. plant from Harvard Forest, originally from plants cultivated by George Newman |
| *Sarracenia alabamensis ssp. alabamensis 1* | ENA | *ITS* | GBANK-JQ218237 | Butler 9 (GH); cultiv. plant from Harvard Forest, originally from plants cultivated by George Newman |
| *Sarracenia alabamensis ssp. alabamensis 1* | ENA | *ITS* | GBANK-JQ218238 | Butler 9 (GH); cultiv. plant from Harvard Forest, originally from plants cultivated by George Newman |
| *Sarracenia alabamensis ssp. alabamensis 1* | ENA | *matK* | GBANK-JQ218245 | Butler 9 (GH); cultiv. plant from Harvard Forest, originally from plants cultivated by George Newman |
| *Sarracenia alabamensis ssp. alabamensis 1* | ENA | *matR* | GBANK-JQ218276 | Butler 9 (GH); cultiv. plant from Harvard Forest, originally from plants cultivated by George Newman |
| *Sarracenia alabamensis ssp. alabamensis 1* | ENA | *PHYC* | GBANK-JQ260413 | Butler 9 (GH); cultiv. plant from Harvard Forest, originally from plants cultivated by George Newman |
| *Sarracenia alabamensis ssp. alabamensis 1* | ENA | *rps3* | GBANK-JQ218291 | Butler 9 (GH); cultiv. plant from Harvard Forest, originally from plants cultivated by George Newman |
| *Sarracenia alabamensis ssp. alabamensis 1* | ENA | *trnS-trnG* | GBANK-JQ218330 | Butler 9 (GH); cultiv. plant from Harvard Forest, originally from plants cultivated by George Newman |
| *Sarracenia alabamensis ssp. alabamensis 2* | ENA | *26S* | GBANK-JQ519378 | Naczi 2672 (NY); U.S.A. Alabama: Autauga Co., cultiv. by F. Case |
| *Sarracenia alabamensis ssp. alabamensis 2* | ENA | *matR* | GBANK-JQ619013 | Naczi 2672 (NY); U.S.A. Alabama: Autauga Co., cultiv. by F. Case |
| *Sarracenia alabamensis ssp. wherryi* | ENA | *26S* | GBANK-DQ076325.1 | GBANK-DQ076325.1 |
| *Sarracenia alata* | ENA | *rps3* | GBANK-JQ218285 | Butler 2 (GH); cultiv. plant from Harvard Forest, originally from seed collected in U.S.A. Mississippi: Stone Co. |
| *Sarracenia alata* | ENA | *ITS* | GBANK-JQ218240 | Butler 25 (GH); cultiv. plant from Harvard Forest, originally from seed collected in U.S.A. Mississippi: Stone Co. |
| *Sarracenia alata* | ENA | *matK* | GBANK-JQ218254 | Butler 25 (GH); cultiv. plant from Harvard Forest, originally from seed collected in U.S.A. Mississippi: Stone Co. |
| *Sarracenia alata* | ENA | *matR* | GBANK-JQ218266 | Butler 25 (GH); cultiv. plant from Harvard Forest, originally from seed collected in U.S.A. Mississippi: Stone Co. |
| *Sarracenia alata* | ENA | *PHYC* | GBANK-JQ260408 | Butler 25 (GH); cultiv. plant from Harvard Forest, originally from seed collected in U.S.A. Mississippi: Stone Co. |
| *Sarracenia alata* | ENA | *psbA-trnH* | GBANK-JQ218309 | Butler 25 (GH); cultiv. plant from Harvard Forest, originally from seed collected in U.S.A. Mississippi: Stone Co. |
| *Sarracenia alata* | ENA | *trnS-trnG* | GBANK-JQ218322 | Butler 25 (GH); cultiv. plant from Harvard Forest, originally from seed collected in U.S.A. Mississippi: Stone Co. |
| *Sarracenia flava* | ENA | *ITS* | GBANK-JQ218236 | Butler 8 (GH); cultiv. plant from Harvard Forest, originally from seed collected in U.S.A. Florida: Walton Co. |
| *Sarracenia flava* | ENA | *matK* | GBANK-JQ218257 | Butler 8 (GH); cultiv. plant from Harvard Forest, originally from seed collected in U.S.A. Florida: Walton Co. |
| *Sarracenia flava* | ENA | *matR* | GBANK-JQ218265 | Butler 8 (GH); cultiv. plant from Harvard Forest, originally from seed collected in U.S.A. Florida: Walton Co. |
| *Sarracenia flava* | ENA | *PHYC* | GBANK-JQ260412 | Butler 8 (GH); cultiv. plant from Harvard Forest, originally from seed collected in U.S.A. Florida: Walton Co. |
| *Sarracenia flava* | ENA | *psbA-trnH* | GBANK-JQ218313 | Butler 8 (GH); cultiv. plant from Harvard Forest, originally from seed collected in U.S.A. Florida: Walton Co. |
| *Sarracenia flava* | ENA | *rps3* | GBANK-JQ218284 | Butler 8 (GH); cultiv. plant from Harvard Forest, originally from seed collected in U.S.A. Florida: Walton Co. |
| *Sarracenia flava* | ENA | *trnS-trnG* | GBANK-JQ218321 | Butler 8 (GH); cultiv. plant from Harvard Forest, originally from seed collected in U.S.A. Florida: Walton Co. |
| *Sarracenia jonesii* | ENA | *matK* | GBANK-JQ218248 | Butler 11 (GH); cultiv. plant from Harvard Forest, originally from outcrossed seeds from cultivated plants grown by David Mellard in Atlanta, GA |
| *Sarracenia jonesii* | ENA | *psbA-trnH* | GBANK-JQ218307 | Butler 11 (GH); cultiv. plant from Harvard Forest, originally from outcrossed seeds from cultivated plants grown by David Mellard in Atlanta, GA |
| *Sarracenia jonesii* | ENA | *rps3* | GBANK-JQ218292 | Butler 11 (GH); cultiv. plant from Harvard Forest, originally from outcrossed seeds from cultivated plants grown by David Mellard in Atlanta, GA |
| *Sarracenia jonesii* | ENA | *PHYC* | GBANK-JQ260405 | Butler 11 (GH); cultiv. plant from Harvard Forest, originally from outcrossed seeds from cultivated plants grown by David Mellard in Atlanta, GA |
| *Sarracenia jonesii* | ENA | *matR* | GBANK-JQ218274 | Butler 11 (GH); cultiv. plant from Harvard Forest, originally from outcrossed seeds from cultivated plants grown by David Mellard in Atlanta, GA |
| *Sarracenia jonesii* | ENA | *trnS-trnG* | GBANK-JQ218331 | Butler 11 (GH); cultiv. plant from Harvard Forest, originally from outcrossed seeds from cultivated plants grown by David Mellard in Atlanta, GA |
| *Sarracenia leucophylla* | ENA | *rps3* | GBANK-JQ218293 | Butler 12 (GH); cultiv. plant from Harvard Forest, originally from seed collected in U.S.A. Florida: Walton Co. |
| *Sarracenia leucophylla* | ENA | *ITS* | GBANK-JQ218241 | Butler 12 (GH); cultiv. plant from Harvard Forest, originally from seed collected in U.S.A. Florida: Walton Co. |
| *Sarracenia leucophylla* | ENA | *matK* | GBANK-JQ218249 | Butler 12 (GH); cultiv. plant from Harvard Forest, originally from seed collected in U.S.A. Florida: Walton Co. |
| *Sarracenia leucophylla* | ENA | *matR* | GBANK-JQ218278 | Butler 12 (GH); cultiv. plant from Harvard Forest, originally from seed collected in U.S.A. Florida: Walton Co. |
| *Sarracenia leucophylla* | ENA | *PHYC* | GBANK-JQ260406 | Butler 12 (GH); cultiv. plant from Harvard Forest, originally from seed collected in U.S.A. Florida: Walton Co. |
| *Sarracenia leucophylla* | ENA | *psbA-trnH* | GBANK-JQ218310 | Butler 12 (GH); cultiv. plant from Harvard Forest, originally from seed collected in U.S.A. Florida: Walton Co. |
| *Sarracenia leucophylla* | ENA | *trnS-trnG* | GBANK-JQ218329 | Butler 12 (GH); cultiv. plant from Harvard Forest, originally from seed collected in U.S.A. Florida: Walton Co. |
| *Sarracenia minor 1* | ENA | *ITS* | GBANK-JQ218234 | Butler 6 (GH); cultiv. plant from Harvard Forest, originally from seed collected in U.S.A. South Carolina: Dorchester Co. |
| *Sarracenia minor 1* | ENA | *matK* | GBANK-JQ218256 | Butler 6 (GH); cultiv. plant from Harvard Forest, originally from seed collected in U.S.A. South Carolina: Dorchester Co. |
| *Sarracenia minor 1* | ENA | *matR* | GBANK-JQ218273 | Butler 6 (GH); cultiv. plant from Harvard Forest, originally from seed collected in U.S.A. South Carolina: Dorchester Co. |
| *Sarracenia minor 1* | ENA | *PHYC* | GBANK-JQ260410 | Butler 6 (GH); cultiv. plant from Harvard Forest, originally from seed collected in U.S.A. South Carolina: Dorchester Co. |
| *Sarracenia minor 1* | ENA | *psbA-trnH* | GBANK-JQ218312 | Butler 6 (GH); cultiv. plant from Harvard Forest, originally from seed collected in U.S.A. South Carolina: Dorchester Co. |
| *Sarracenia minor 1* | ENA | *rps3* | GBANK-JQ218289 | Butler 6 (GH); cultiv. plant from Harvard Forest, originally from seed collected in U.S.A. South Carolina: Dorchester Co. |
| *Sarracenia minor 1* | ENA | *trnS-trnG* | GBANK-JQ218327 | Butler 6 (GH); cultiv. plant from Harvard Forest, originally from seed collected in U.S.A. South Carolina: Dorchester Co. |
| *Sarracenia minor 2* | ENA | *26S* | GBANK-JQ519383 | Naczi 2664 (NY); U.S.A. Georgia: Charlton Co., cultiv. by F. Case |
| *Sarracenia oreophila 1* | ENA | *ITS* | GBANK-JQ218235 | Butler 7 (GH); cultiv. plant from Harvard Forest, originally from plants cultivated by George Newman |
| *Sarracenia oreophila 1* | ENA | *matK* | GBANK-JQ218244 | Butler 7 (GH); cultiv. plant from Harvard Forest, originally from plants cultivated by George Newman |
| *Sarracenia oreophila 1* | ENA | *matR* | GBANK-JQ218275 | Butler 7 (GH); cultiv. plant from Harvard Forest, originally from plants cultivated by George Newman |
| *Sarracenia oreophila 1* | ENA | *PHYC* | GBANK-JQ260411 | Butler 7 (GH); cultiv. plant from Harvard Forest, originally from plants cultivated by George Newman |
| *Sarracenia oreophila 1* | ENA | *psbA-trnH* | GBANK-JQ218301 | Butler 7 (GH); cultiv. plant from Harvard Forest, originally from plants cultivated by George Newman |
| *Sarracenia oreophila 1* | ENA | *rps3* | GBANK-JQ218290 | Butler 7 (GH); cultiv. plant from Harvard Forest, originally from plants cultivated by George Newman |
| *Sarracenia oreophila 1* | ENA | *trnS-trnG* | GBANK-JQ218328 | Butler 7 (GH); cultiv. plant from Harvard Forest, originally from plants cultivated by George Newman |
| *Sarracenia oreophila 2* | ENA | *26S* | GBANK-JQ519384 | Naczi 2665 (NY); U.S.A. Georgia: Towns Co., cultiv. by F. Case |
| *Sarracenia oreophila 2* | ENA | *matR* | GBANK-JQ619019 | Naczi 2665 (NY); U.S.A. Georgia: Towns Co., cultiv. by F. Case |
| *Sarracenia psittacina* | ENA | *ITS* | GBANK-JQ218239 | Butler 16 (GH); cultiv. plant from California Carnivores, Sebastopol, CA |
| *Sarracenia psittacina* | ENA | *matK* | GBANK-JQ218253 | Butler 16 (GH); cultiv. plant from California Carnivores, Sebastopol, CA |
| *Sarracenia psittacina* | ENA | *psbA-trnH* | GBANK-JQ218308 | Butler 16 (GH); cultiv. plant from California Carnivores, Sebastopol, CA |
| *Sarracenia psittacina* | ENA | *trnS-trnG* | GBANK-JQ218332 | Butler 16 (GH); cultiv. plant from California Carnivores, Sebastopol, CA |
| *Sarracenia psittacina* | ENA | *PHYC* | GBANK-JQ260407 | Butler 16 (GH); cultiv. plant from California Carnivores, Sebastopol, CA |
| *Sarracenia psittacina* | ENA | *matR* | GBANK-JQ218272 | Butler 16 (GH); cultiv. plant from California Carnivores, Sebastopol, CA |
| *Sarracenia purpurea ssp. purpurea 1* | ENA | *ITS* | GBANK-JQ218232 | Butler 3 (GH); U.S.A. Massachusetts: Worcester Co., wild coll. |
| *Sarracenia purpurea ssp. purpurea 1* | ENA | *matK* | GBANK-JQ218247 | Butler 3 (GH); U.S.A. Massachusetts: Worcester Co., wild coll. |
| *Sarracenia purpurea ssp. purpurea 1* | ENA | *matR* | GBANK-JQ218267 | Butler 3 (GH); U.S.A. Massachusetts: Worcester Co., wild coll. |
| *Sarracenia purpurea ssp. purpurea 1* | ENA | *psbA-trnH* | GBANK-JQ218304 | Butler 3 (GH); U.S.A. Massachusetts: Worcester Co., wild coll. |
| *Sarracenia purpurea ssp. purpurea 1* | ENA | *rps3* | GBANK-JQ218286 | Butler 3 (GH); U.S.A. Massachusetts: Worcester Co., wild coll. |
| *Sarracenia purpurea ssp. purpurea 1* | ENA | *trnS-trnG* | GBANK-JQ218320 | Butler 3 (GH); U.S.A. Massachusetts: Worcester Co., wild coll. |
| *Sarracenia purpurea ssp. purpurea 2* | ENA | *ITS* | GBANK-JQ218231 | Butler 13 (GH); cultiv. plant from California Carnivores, Sebastopol, CA |
| *Sarracenia purpurea ssp. purpurea 2* | ENA | *matK* | GBANK-JQ218250 | Butler 13 (GH); cultiv. plant from California Carnivores, Sebastopol, CA |
| *Sarracenia purpurea ssp. purpurea 2* | ENA | *matR* | GBANK-JQ218269 | Butler 13 (GH); cultiv. plant from California Carnivores, Sebastopol, CA |
| *Sarracenia purpurea ssp. purpurea 2* | ENA | *PHYC* | GBANK-JQ260402 | Butler 13 (GH); cultiv. plant from California Carnivores, Sebastopol, CA |
| *Sarracenia purpurea ssp. purpurea 2* | ENA | *psbA-trnH* | GBANK-JQ218305 | Butler 13 (GH); cultiv. plant from California Carnivores, Sebastopol, CA |
| *Sarracenia purpurea ssp. purpurea 2* | ENA | *trnS-trnG* | GBANK-JQ218323 | Butler 13 (GH); cultiv. plant from California Carnivores, Sebastopol, CA |
| *Sarracenia purpurea ssp. purpurea 3* | ENA | *26S* | GBANK-JQ519385 | Naczi 1400 (NY); U.S.A. Michigan: Cheboygan Co., wild coll. |
| *Sarracenia purpurea ssp. purpurea 3* | ENA | *matR* | GBANK-JQ619020 | Naczi 1400 (NY); U.S.A. Michigan: Cheboygan Co., wild coll. |
| *Sarracenia purpurea ssp. venosa var. montana* | ENA | *ITS* | GBANK-JQ218230 | no voucher; cultiv. plant from Meadowview Biological Research Station, Woodford, VA |
| *Sarracenia purpurea ssp. venosa var. montana* | ENA | *matK* | GBANK-JQ218246 | no voucher; cultiv. plant from Meadowview Biological Research Station, Woodford, VA |
| *Sarracenia purpurea ssp. venosa var. montana* | ENA | *matR* | GBANK-JQ218268 | no voucher; cultiv. plant from Meadowview Biological Research Station, Woodford, VA |
| *Sarracenia purpurea ssp. venosa var. montana* | ENA | *PHYC* | GBANK-JQ260401 | no voucher; cultiv. plant from Meadowview Biological Research Station, Woodford, VA |
| *Sarracenia purpurea ssp. venosa var. montana* | ENA | *psbA-trnH* | GBANK-JQ218300 | no voucher; cultiv. plant from Meadowview Biological Research Station, Woodford, VA |
| *Sarracenia purpurea ssp. venosa var. montana* | ENA | *trnS-trnG* | GBANK-JQ218319 | no voucher; cultiv. plant from Meadowview Biological Research Station, Woodford, VA |
| *Sarracenia purpurea ssp. venosa var. venosa* | ENA | *matK* | GBANK-JQ218251 | Butler 14 (GH); cultiv. plant from California Carnivores, Sebastopol, CA |
| *Sarracenia purpurea ssp. venosa var. venosa* | ENA | *matR* | GBANK-JQ218270 | Butler 14 (GH); cultiv. plant from California Carnivores, Sebastopol, CA |
| *Sarracenia purpurea ssp. venosa var. venosa* | ENA | *PHYC* | GBANK-JQ260403 | Butler 14 (GH); cultiv. plant from California Carnivores, Sebastopol, CA |
| *Sarracenia purpurea ssp. venosa var. venosa* | ENA | *psbA-trnH* | GBANK-JQ218306 | Butler 14 (GH); cultiv. plant from California Carnivores, Sebastopol, CA |
| *Sarracenia purpurea ssp. venosa var. venosa* | ENA | *rps3* | GBANK-JQ218287 | Butler 14 (GH); cultiv. plant from California Carnivores, Sebastopol, CA |
| *Sarracenia purpurea ssp. venosa var. venosa* | ENA | *trnS-trnG* | GBANK-JQ218324 | Butler 14 (GH); cultiv. plant from California Carnivores, Sebastopol, CA |
| *Sarracenia rosea 1* | ENA | *matK* | GBANK-JQ218252 | Butler 15 (GH); cultiv. plant from California Carnivores, Sebastopol, CA |
| *Sarracenia rosea 1* | ENA | *matR* | GBANK-JQ218271 | Butler 15 (GH); cultiv. plant from California Carnivores, Sebastopol, CA |
| *Sarracenia rosea 1* | ENA | *psbA-trnH* | GBANK-JQ218303 | Butler 15 (GH); cultiv. plant from California Carnivores, Sebastopol, CA |
| *Sarracenia rosea 1* | ENA | *trnS-trnG* | GBANK-JQ218325 | Butler 15 (GH); cultiv. plant from California Carnivores, Sebastopol, CA |
| *Sarracenia rosea 1* | ENA | *PHYC* | GBANK-JQ260404 | Butler 15 (GH); cultiv. plant from California Carnivores, Sebastopol, CA |
| *Sarracenia rosea 2* | ENA | *matR* | GBANK-JQ619026 | Naczi 2671 (NY); U.S.A. Alabama: Mobile Co., cultiv. by F. Case |
| *Sarracenia rubra ssp. gulfensis* | ENA | *26S* | GBANK-DQ076326.1 | GBANK-DQ076326.1 |
| *Sarracenia rubra ssp. rubra* | ENA | *matR* | GBANK-JQ218277 | Butler 5 (GH); cultiv. plant from Harvard Forest, originally from outcrossed seeds from cultivated plants grown by David Mellard in Atlanta, GA |
| *Sarracenia rubra ssp. rubra* | ENA | *PHYC* | GBANK-JQ260409 | Butler 5 (GH); cultiv. plant from Harvard Forest, originally from outcrossed seeds from cultivated plants grown by David Mellard in Atlanta, GA |
| *Sarracenia rubra ssp. rubra* | ENA | *psbA-trnH* | GBANK-JQ218311 | Butler 5 (GH); cultiv. plant from Harvard Forest, originally from outcrossed seeds from cultivated plants grown by David Mellard in Atlanta, GA |
| *Sarracenia rubra ssp. rubra* | ENA | *rps3* | GBANK-JQ218288 | Butler 5 (GH); cultiv. plant from Harvard Forest, originally from outcrossed seeds from cultivated plants grown by David Mellard in Atlanta, GA |
| *Sarracenia rubra ssp. rubra* | ENA | *trnS-trnG* | GBANK-JQ218326 | Butler 5 (GH); cultiv. plant from Harvard Forest, originally from outcrossed seeds from cultivated plants grown by David Mellard in Atlanta, GA |
| *Sarracenia rubra ssp. rubra* | ENA | *ITS* | GBANK-JQ218233 | Butler 5 (GH); cultiv. plant from Harvard Forest, originally from outcrossed seeds from cultivated plants grown by David Mellard in Atlanta, GA |
| *Sarracenia rubra ssp. rubra* | ENA | *matK* | GBANK-JQ218255 | Butler 5 (GH); cultiv. plant from Harvard Forest, originally from outcrossed seeds from cultivated plants grown by David Mellard in Atlanta, GA |
